# Group learning capacity: the roles of open-mindedness and shared vision

**DOI:** 10.3389/fpsyg.2015.00150

**Published:** 2015-02-27

**Authors:** Mimi Lord

**Affiliations:** Weatherhead School of Management, Case Western Reserve UniversityCleveland, OH, USA

**Keywords:** learning capacity, shared vision, open-mindedness, organizational learning, absorptive capacity, cohesiveness, task conflict, groupthink

## Abstract

Open-mindedness (OPM) is a construct that is considered a key foundational aspect of learning in individuals, groups and organizations. Also known as critical inquiry or reflection, OPM is believed to increase learning through examination of prior beliefs, decisions and mistakes, and also through openness to new ideas. Renowned theorists including Dewey and Argyris have emphasized the relationship between OPM and learning, yet little quantitative research has tested it or examined moderators of the linkage. The setting for the current study is that of endowment investment committees at U.S. universities and colleges who need to make knowledgeable and well-reasoned decisions about the composition of investment portfolios. Findings indicate that OPM has a positive, significant effect on group learning capacity (LCAP) and also that shared vision, which represents the group's collective purpose and direction, moderates that relationship. The literature review and discussion offer insights about how OPM is related to the research on group conflict, and how shared vision (SHV) differs from concepts such as interpersonal cohesiveness and conformity that have been associated with groupthink. A review of relevant research from the fields of organizational learning, group dynamics, and absorptive capacity provides context for the development of the hypotheses and the discussion of findings.

## Introduction

From Socrates to modern learning theorists, open-mindedness (OPM) has been considered essential to learning and understanding. Dewey (1933), Kolb (1984) and Argyris (1976) all have underscored the significance that the ancient Greeks placed on inquiry, openness, dialog, and critical reflection. Yet empirical research examining the relationship between OPM and learning is scant relative to the volumes on theory, and particularly in group decision-making domains. In addition, little empirical research has explored how interpersonal dynamics in groups might moderate the relationship between OPM and learning.

The purpose of this study, situated within the broad fields of organizational learning and behavior, is to provide additional insights about how groups learn and especially about the types of dialogs and group dynamics that foster learning. We will test the relationship between OPM and learning capacity (LCAP) in decision-making groups, as well as the effect of shared vision (SHV) on that relationship. In our literature review and discussion, we will explore how different types of cohesiveness affect group learning and effectiveness, and whether SHV is situated within the cohesiveness spectrum. We will argue that because SHV coexists with OPM in this study, it provides a positive contribution to group learning capacity. In contrast, if SHV co-existed with closed-mindedness, one could expect it (along with closed-mindedness) to detract from group learning capacity. In the latter context, SHV might be related to the strong interpersonal attraction aspect of cohesiveness associated with groupthink, a type of behavior that lacks independent critical thinking and is focused primarily on reaching consensus (Janis, 1972, 1983), thus restricting group learning capacity.

Given that OPM and group task conflict overlap with regards to the emphasis placed on critical reflection and assessment, we suggest that literature on task conflict has strong relevance to this study. Task conflict occurs when there are “disagreements among group members about the content of the tasks being performed, including differences in viewpoints, ideas, and opinions” (Jehn, 1995, p. 258). We will discuss similarities between OPM and task conflict, and especially when expressions of task conflict are mild rather than intense (Todorova et al., 2014). While task conflict has been researched predominantly in terms of its positive influence on decision quality, the current study uses the concept of OPM as a predictor and examines its influence on a different dependent variable, learning capacity.

We adopt the term LCAP as our dependent variable because it signifies the ability of organizations, groups/teams and individuals to engage in learning processes leading to positive outcomes such as performance, competitive advantage, innovation, adaptability, and knowledge transfer (Volberda et al., 2010; Van Wijk et al., 2011). The term has been used as a synonym for “absorptive capacity” to describe a group's ability to acquire relevant external information, integrate it with existing knowledge, and exploit it to commercial benefit (Cohen and Levinthal, 1990). LCAP has been characterized as a process of gaining knowledge (Lane et al., 2006), which is considered a key resource of an organization and a primary source of competitive advantage (Barney, 1991; Grant, 1996; Kogut and Zander, 1996).

We define OPM as a group's critical assessment of its assumptions, beliefs and prior actions, as well as its openness to new ideas (Sinkula et al., 1997; Calantone et al., 2002). This critical assessment concept resembles Dewey's description of reflection: “assessing the grounds (justification) of one's beliefs” (Dewey, 1933, p. 9). Reflection is often used as a synonym for higher-order processes (Mezirow, 1990) or double-loop learning (Argyris and Schön, 1978), which results in transcending current ways of thinking and acting. The OPM construct in this study also bears strong resemblance to the term “authentic inquiry” (Mazutis and Slawinski, 2008), which encourages critical reflection and open dialog. Without open dialog, individuals may engage in defensive routines that inhibit their learning (Argyris and Schön, 1978). They may not be willing to examine and learn from past mistakes and thus may withhold information that they perceive as detrimental to others' perceptions of themselves. When authentic dialog is encouraged, members are more likely to confront conflict through inquiry and to seek understanding without engaging in power struggles (Mazutis and Slawinski, 2008).

The OPM construct has been employed widely in marketing literature as a first-order factor within a second-order reflective factor called learning orientation, which is described as a set of organizational values that influence individuals' and groups' propensity to seek and use knowledge (Sinkula et al., 1997). Organizations with a learning orientation have a sense of direction for their learning as well as a critical-assessment approach that encourages open debates and questioning of assumptions (Slater and Narver, 1995). Learning orientation studies typically include learning commitment and SHV as other first-order factors. To the best of our knowledge, the current study is unique in focusing on OPM and SHV as stand-alone factors that influence learning capacity. We employ OPM as having the main effect on LCAP due to its prominence in theoretical literature, and SHV as a moderator due to its motivational and purpose-oriented characteristics that would enhance the primary relationship. Learning commitment is not included in this study since we believe that its characteristics are largely subsumed in SHV and OPM.

Unlike other studies on LCAP and its antecedents in the domains of manufacturing, marketing and information technology, this study's domain is that of decision-making committees in non-profit institutions, and specifically the investment committees of college and university endowments. These committees, composed largely of alumni volunteers, typically are charged with making important decisions affecting the composition and performance of endowment portfolios. Understanding factors that affect portfolio decisions and performance is critical for college and university leaders since the endowment earnings can have a significant impact on the financial health of the institution (Brown et al., 2010). As with many other decision-making groups whose environments are constantly changing, investment committees need to be able to acquire relevant information from the external world (i.e., the financial markets and external experts) on a continual basis, to assimilate it with their existing knowledge, and to implement it successfully. In a quantitative study about endowment management (Lord, 2014b), committees who understood how to implement their investment-related knowledge had more diversified portfolios and higher risk-adjusted returns.

In the following section, we will formalize our hypotheses by examining research on learning capacity, OPM and shared vision. Our empirical study is based on a survey of “key informants” who are involved with investment committees at 168 U.S. university endowments.

## Background and hypotheses

Our study is situated in the field of organizational learning, which has been defined as a process of improving organizational actions through better knowledge and understanding (Fiol and Lyles, 1985; Garvin, 1993). Organizational learning researchers have addressed cognitive types of learning (Kolb, 1984; Argyris, 1999) as well as learning processes (Levitt and March, 1988; Huber, 1991; Tippins and Sohi, 2003). Certain researchers (Huber, 1991; Tippins and Sohi, 2003) refer to four processes in organizational learning: (a) information acquisition; (b) information sharing; (c) information interpretation; and (d) information storage. Other organizational learning researchers refer to only two processes: (a) explore and exploit (March, 1991); (b) organizational search and trial/error (Levitt and March, 1988); and (c) reflection and action (Edmondson, 2002). Learning theorists differ as to whether taking action (or exploiting) is a requirement of organizational learning. Huber (1991) and Tippins and Sohi (2003) clearly do not have that requirement. In fact, Huber states that organizational learning has occurred if, through the group's processing of information, the range of its *potential* behaviors has changed. In contrast, Edmondson (2002), March (1991) and Levitt and March (1988) clearly require that action must be taken in order for learning to have occurred.

Another stream of research related to the field of organizational learning is called “knowledge management” (Bassi, 1999), which focuses largely on managing what is learned, including storing and retrieving knowledge. Also related is the dynamic capabilities framework, developed by Teece et al. (1997), which refers to the ability to renew and adapt competencies in order to be in sync with rapidly changing business environments.

While incorporating aspects of these related constructs, LCAP is distinguished by its emphasis on acquiring relevant “external” information and by its imperative of implementing or “exploiting” the knowledge successfully. LCAP has been theorized and employed in research studies as having one, two, three or four dimensions (or processes). In early conceptualizations of the learning (or absorptive) capacity construct, Cohen and Levinthal (1989, 1990) referred to its three dimensions of identifying relevant information, assimilating it, and applying new knowledge successfully, yet they did not provide a measurement tool other than research and development expenditures. Szulanski (1996) used a unidimensional measure and found that the lack of recipient absorptive capacity is a major barrier to knowledge transfer between different functions in an organization. Zahra and George (2002) re-conceptualized the construct into two primary dimensions with each having two sub-dimensions: potential absorptive capacity consisting of acquisition and assimilation of new external knowledge; and realized absorptive capacity consisting of knowledge transformation and exploitation. Jansen et al. (2005) operationalized the construct with all four sub-dimensions and tested for antecedents of coordination, systems, and socialization capabilities. Lichtenthaler (2009) followed Cohen and Levinthal's guidance of three dimensions, employing exploratory, transformative, and exploitative learning processes with measurement items borrowed from previous studies. In sum, the LCAP construct has been operationalized in multiple ways with varying dimensions and scales (Lane et al., 2006). In this study we are focused on the holistic meaning of LCAP and not on the distinct dimensions or processes of it. Therefore, we employ a unidimensional factor for LCAP that we believe captures Cohen and Levinthal's (1989, 1990) conceptualization.

Our hypotheses in this study are in alignment with: (a) the learning disciplines of Senge (1990) that emphasize the need for SHV and open dialogs that are oriented to finding truth; (b) a set of learning-oriented activities called “teaming” which encourage group members to collaborate and to engage in honest and reflective conversations (Edmondson, 2012); and (c) a learning environment called “ba” which supports learning creation and an ongoing re-evaluation of existing premises (Nonaka et al., 2000).

## Open-mindedness and learning capacity

Dewey (1933) stated that OPM (which he called “reflection”) refers to assessing the grounds or justification of one's beliefs. Similarly, more recent researchers argue that OPM is critical for examining individuals' mental models, which are deeply held beliefs or conceptions that may confine them to familiar patterns of thinking and acting (Senge, 1990; Day and Nedungadi, 1994; Sinkula et al., 1997). If these deeply held beliefs and assumptions are not questioned and altered, groups' effectiveness will be diminished (Day, 1994; Sinkula, 1994). When group members have differences in their interpretation of task-related issues, they experience greater learning and gain a more accurate assessment of situations (Fiol, 1994). Argyris and Schön (1978) maintain that a key aspect of OPM is its attention to detecting and correcting errors, which they consider essential to organizational learning.

Examination of deeply held convictions and consideration of alternative perspectives often involve a relatively high level of disagreement (Janis, 1972; Jehn, 1995; Slater and Narver, 1995). Disagreement that remains task-oriented is referred to as both “cognitive conflict” and “task conflict” and has been found to result in higher-quality decisions (Amason and Schweiger, 1994; Amason, 1996). In their research on corporate board decision-making, Forbes and Milliken (1999) argued that cognitive conflict fosters an environment that is characterized by a task-oriented focus and a tolerance of multiple viewpoints and opinions; thus, it promotes critical discussions and helps to prevent groupthink. Because cognitive conflict remains task-oriented, it is not to be confused with affective (or relationship) conflict, which can become personal and damage the group's commitment and ability to work together (Amason, 1996). Researchers have suggested techniques and tools to help leaders and group members foster and maintain OPM so that conflicts remain task-oriented and not personal. Among those are: (a) developing and expressing one's own view; (b) questioning and understanding other views; (c) integrating and creating solutions; and (d) agreeing to and implementing solutions (Tjosvold et al., 2014). Another suggestion is to assign a member (or members) to serve as a devil's advocate, questioning group members' underlying assumptions and opinions (Amason, 1996).

Cognitive (or task) conflict has typically been studied as an antecedent to higher quality decisions rather than to learning capacity. One empirical study found that “openness” led to organizational learning (Hult et al., 2000) but the openness construct had two dimensions, participativeness and reflectiveness, whereas only the latter resembles the OPM construct employed in this study. As noted previously, OPM has been used in empirical studies more as a first-order factor of learning orientation than as a stand-alone construct. In a study that did examine it as a stand-alone factor, OPM was found to have a significant and positive effect on product innovation (Calisir et al., 2013); the study did not employ a learning construct. Although a significant body of literature has *discussed* the linkage between OPM and learning, we have been unable to find a study that *empirically tests* that relationship in group decision-making settings.

*Hypothesis 1. Open-mindedness will have a positive effect on learning capacity*.

## Shared vision as a moderator

SHV has been described as the embodiment of a group's collective goals and aspirations (Tsai and Ghoshal, 1998) as well as its shared sense of purpose and operating values (Senge, 1990). SHV is considered essential for proactive learning because it fosters commitment, energy and purpose among group members (Tobin, 1993; Day, 1994). Similarly, Senge (1990) states that learning cannot occur without SHV since it provides the “pull” toward goals that helps to overcome forces of inertia.

SHV helps to motivate teams (Van den Bossche et al., 2006); to promote sharing of perspectives and knowledge (Bunderson and Reagans, 2010); to promote positive feelings and commitment among members (Boyatzis, 2008); to foster greater organizational engagement (Mahon et al., 2014); and to legitimize the acquisition and assessment of new knowledge (Lyles and Salk, 1996). When team members share common or cooperative goals they are open to problem-solving approaches that help them learn from mistakes (Tjosvold et al., 2004); in contrast, competitive goals have been found to correlate negatively with collective problem-solving approaches and to undermine group learning. Tsai and Ghoshal (1998) state that SHV and collective goals are reflections of the cognitive dimension of social capital.

Strong interpersonal cohesiveness of group members, on the other hand, has been associated with groupthink (Mullen et al., 1994), which has been described as a dysfunctional mode of decision making that can occur when there is a lack of independent critical thinking and when there is a strong desire to have unanimity among members (Janis, 1972, 1983). However, while cohesiveness may be a determinant of groupthink, it is not sufficient (Janis, 1972). Cohesiveness must be accompanied by directive leadership and a lack of cognitive conflict to foster groupthink; when cognitive conflict is present it fosters an environment with a task-oriented focus and a tolerance of multiple viewpoints and opinions (Janis, 1983; Bernthal and Insko, 1993). Thus, a distinction has been made between a type of cohesiveness that is task-oriented and a type that is focused on interpersonal attraction, with only the latter being linked to groupthink (Hogg, 1993). This view was supported in a quantitative study by Mullen et al. (1994): interpersonal attraction contributed to groupthink and poor decision quality, whereas commitment to task tended to ward it off. Researchers also have studied the possible relationship between conformity and groupthink, and particularly when there is a strong “compliance” aspect to conformity. Compliance refers to situations where group members are in agreement publicly but are not in agreement privately; this can occur when members suppress their private doubts about the group decision for reasons such as fear of recrimination if they were to dissent (McCauley, 1989).

Our argument in the current study is that SHV is about collective purpose, goals and tasks that increase the effect of OPM on learning capacity. In this study, SHV is not driven by a desire to be unanimous due to either strong interpersonal attraction or compliance motives that have been associated with groupthink. Thus, it seems logical that SHV would provide the beneficial effect of keeping open-minded dialogs on a collective learning track that supports the group's goals.

*Hypothesis 2. Shared vision will strengthen the positive effect of open-mindedness on learning capacity*.

**Figure 1:**
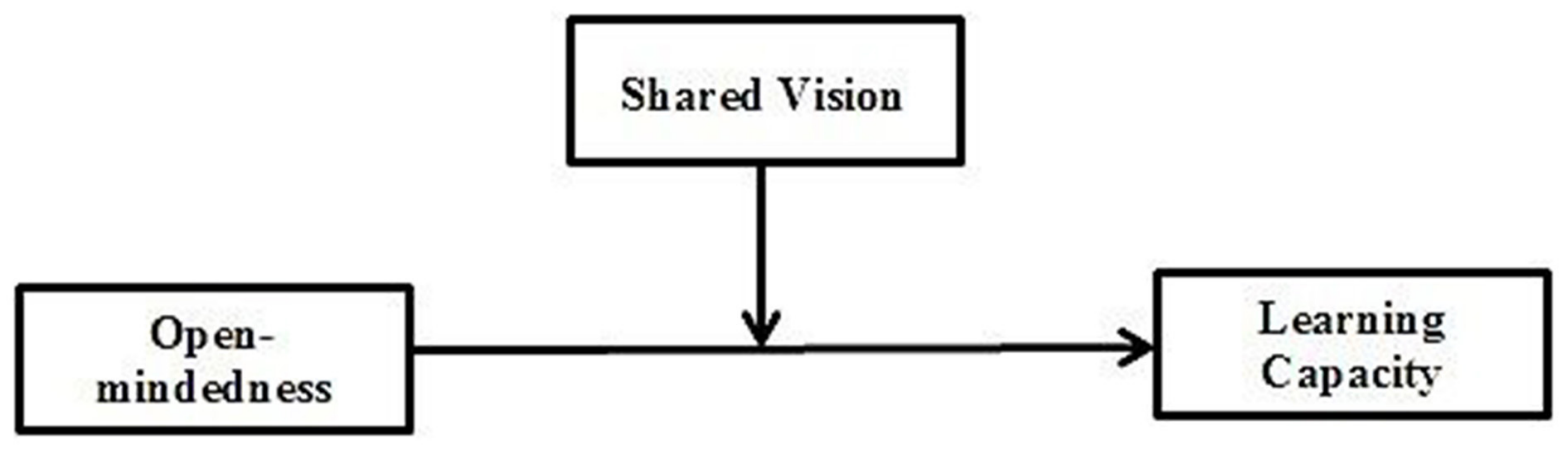
**Hypothesized model**.

Figure 1 shows the hypothesized model, with SHV moderating the effect of OPM on learning capacity.

## Data collection, screening and sample

Empirical data to test the hypothesized relationships were obtained by an electronic survey. Emails soliciting participation were sent to 650 colleges and universities, all of which had participated in the 2009 endowment survey by the National Association of College and University Business Officers (NACUBO) and Commonfund (2009–2010), or in previous annual surveys sponsored solely by NACUBO. Non-members of NACUBO may purchase a version of the 2009 study at http://www.nacubo.org/Products/Online_Research_Products/2009_NACUBO_Commonfund_Study_of_Endowments.html. Emails were addressed to financial officers requesting survey participation by a “key informant”: someone who had regularly attended investment committee meetings for at least several years and was very familiar with the committee's composition, responsibilities, nature of discussions, and decision-making practices. The solicitation email suggested that either the university financial officer most involved with the endowment or the investment committee chair would be an ideal respondent. The Institutional Review Board at the author's institution approved the Informed Consent and ethical conduct of the study, and all protocols governing the use of human subjects were followed. After the initial email of solicitation in September 2010, three reminders were e-mailed over the subsequent 3–4 weeks. Since the questions in the survey related to a period that ended more than a year earlier (June 30, 2009), we were not concerned with slight differences in survey response dates.

A total of 191 colleges/universities responded to the survey; the usable number was reduced to 168, or 25.8%, after eliminating nine cases due to incomplete surveys, three outliers, and 11 institutions for which certain objective data were not available from NACUBO studies. The three outliers had Cook's Distance values greater than 1.0, the threshold suggested as being problematic by Tabachnick and Fidell (2007, p. 75). To determine if the sample was representative of the 650 colleges with five-year performance data in the 2009 NACUBO–Commonfund survey, we conducted an independent samples *t*-test of the means of the five-year annualized performance returns. No significant difference was observed between the means (*t* = −0.656; *df* = 815; *p* = 0.512). The mean return from the NACUBO–Commonfund study was 2.70%, *s* = 2.55%, while the mean of this sample was 2.56%; *s* = 2.10%.

All but four respondents were finance, foundation, or investment officers at their colleges or universities; two were outsourced chief investment officers and two were investment committee members. On average, respondents had served 11 years in an endowment-related role with the college/university. Respondents were from both public (39%) and private (61%) institutions and the size of endowments spanned all six categories in the annual NACUBO–Commonfund study, from less than $25 million to greater than $1 billion. The average endowment size of survey participants as of fiscal year-end 2009 was $315 million, compared to $306 million in the 2009 NACUBO–Commonfund study.

The investment committees in our sample play important roles in key decisions concerning the management of the endowment portfolio. Approximately two-thirds of respondents indicated that the committee makes final decisions about hiring/firing managers and consultants, as well as policy asset allocations.

## Measurement

The full questionnaire included more than 70 items including those relating to factors for the structural model in this study as well as other data about governance issues, staffing, performance and asset allocation. Certain factors that are not included in this study were used in a previous paper about group factors leading to diversified investment portfolios and superior financial performance (Lord, 2014b); information about some of those other factors is included later in this paper in the section called Other Findings. For our model in the current study, we used the items for the latent factors of OPM, SHV and learning capacity. For control variables we used staff size and committee meeting frequency as they were said to relate to learning and performance in a qualitative study of endowments (Lord, 2014a).

## Independent and interaction variables

The scale items for all latent factors employed a 7-point Likert scale ranging from Very Strongly Disagree to Very Strongly Agree; they are provided in the appendix. Items for the independent variable (OPM) and the interaction moderator (shared vision) were adopted from existing scales (Sinkula et al., 1997; Calantone et al., 2002). An example of the items in the OPM construct was, “The committee was not afraid to reflect critically on investment-related assumptions it made,” and a sample item from the SHV construct was, “Our committee was in agreement about the endowment's purpose.”

## Dependent variable

The LCAP scale was developed and adapted from research in the field of absorptive capacity: Jaworski and Kohli (1993); Zahra and George (2002); Jansen et al. (2005), and Szulanski (1996). Items included: “The committee collected in-depth information that was relevant to our investment decisions,” and “The committee knew how to implement new investment knowledge.”

## Factor analysis

Sampling adequacy is excellent with a reading of 0.926 for the Kaiser-Meyer-Olkin Measure of Sampling. Bartlett's test of Sphericity is significant at the 0.000 level, indicating that there are correlations in the data set that are appropriate for factor analysis. Exploratory factor analysis (EFA) was conducted simultaneously with all the items for the latent factors using principal axis factoring with Promax rotation. The purpose of EFA was to determine if the observed variables loaded together as expected, were adequately correlated, and met the criteria of reliability and validity. Three latent factors were clearly observed with sufficient item loadings on each and with minimal cross-loadings. The EFA included the eigenvalues of 11.213 for learning capacity, 2.346 for OPM and 1.348 for shared vision. We assessed scale reliability for each latent factor with Cronbach's alpha, a measure of internal consistency or the closeness of the items for each factor. The Cronbach's alpha is high for all three factors: OPM (0.871), SHV (0.904), and LCAP (0.939), indicating high internal consistency.

EFA was followed by confirmatory factor analysis (CFA) for more rigorous testing and validation of the factor structure. We computed composite reliability (CR) scores for each factor, which were above the minimum threshold of 0.700. CR was 0.860 for OPM, 0.919 for shared vision, and 0.939 for learning capacity. Convergent validity was tested by calculating the average variance extracted (AVE); all factors had an AVE above the recommended threshold of 0.500 (Kline, 2011). Next, we tested discriminant validity by reviewing the maximum shared variance (MSV) and the average shared variance (ASV) for each factor and confirmed that they were less than the AVE for each factor. Discriminant validity was also confirmed in that the square root of the AVE was greater than the inter-factor correlations (Fornell and Larcker, 1981). See Table 1 for details on these measures; square root of the AVE is on the diagonal.

**Table 1:** **Convergent and discriminant validity and reliability**.

	**CR**	**AVE**	**MSV**	**ASV**	**OPM**	**SHV**	**LCAP**
OPM	0.860	0.605	0.598	0.538	**0.778**		
SHV	0.919	0.741	0.477	0.371	0.691	**0.861**	
LCAP	0.939	0.634	0.598	0.431	0.773	0.514	**0.796**

The goodness of fit statistics for the measurement model are shown in Table 2 along with the “ideal thresholds” outlined by Hu and Bentler (1999). Model fit is acceptable in that all ideal thresholds are met except for root mean square error of approximation (RMSEA) which is extremely close at 0.061; other research (Steiger, 2007) stipulates an upper RMSEA limit of 0.07 for acceptable fit.

**Table 2:** **Fit statistics for measurement model**.

**Metric**	**Observed value**	**Ideal threshold**
CMIN/df	1.622	Between 1 and 3
CFI	0.972	>0.950
RMSEA	0.061	<0.060
PCLOSE	0.137	>0.050
SRMR	0.0465	<0.080

Because items for our study's three latent factors were collected via the same instrument at the same time, it was prudent to conduct a common method bias test. We used the common latent factor (CLF) method advocated by MacKenzie and Podsakoff (2012) when no theoretically driven marker variable is collected. We compared the standardized regression weights before and after adding the CLF and found that the differences were all less than 0.200, thus indicating that the model does not suffer from common method bias.

## Results

**Figure 2:**
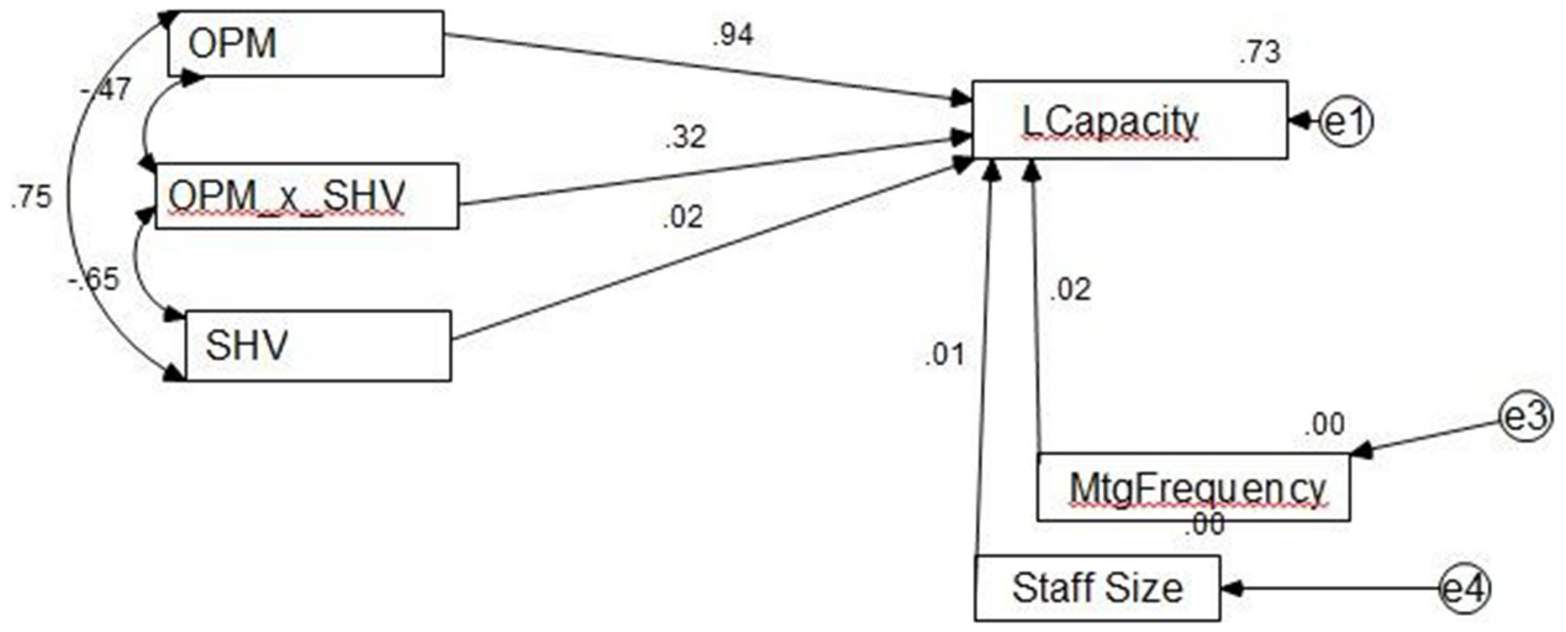
**Structural model results**.

Hypotheses were tested using covariance-based structural equation modeling (SEM) with IBM's AMOS program. Hypothesis 1 is supported in that the standardized regression weight from OPM to LCAP is positive and significant at the 0.001 level. The model with standardized regression weights is shown in Figure 2.

**Figure 3:**
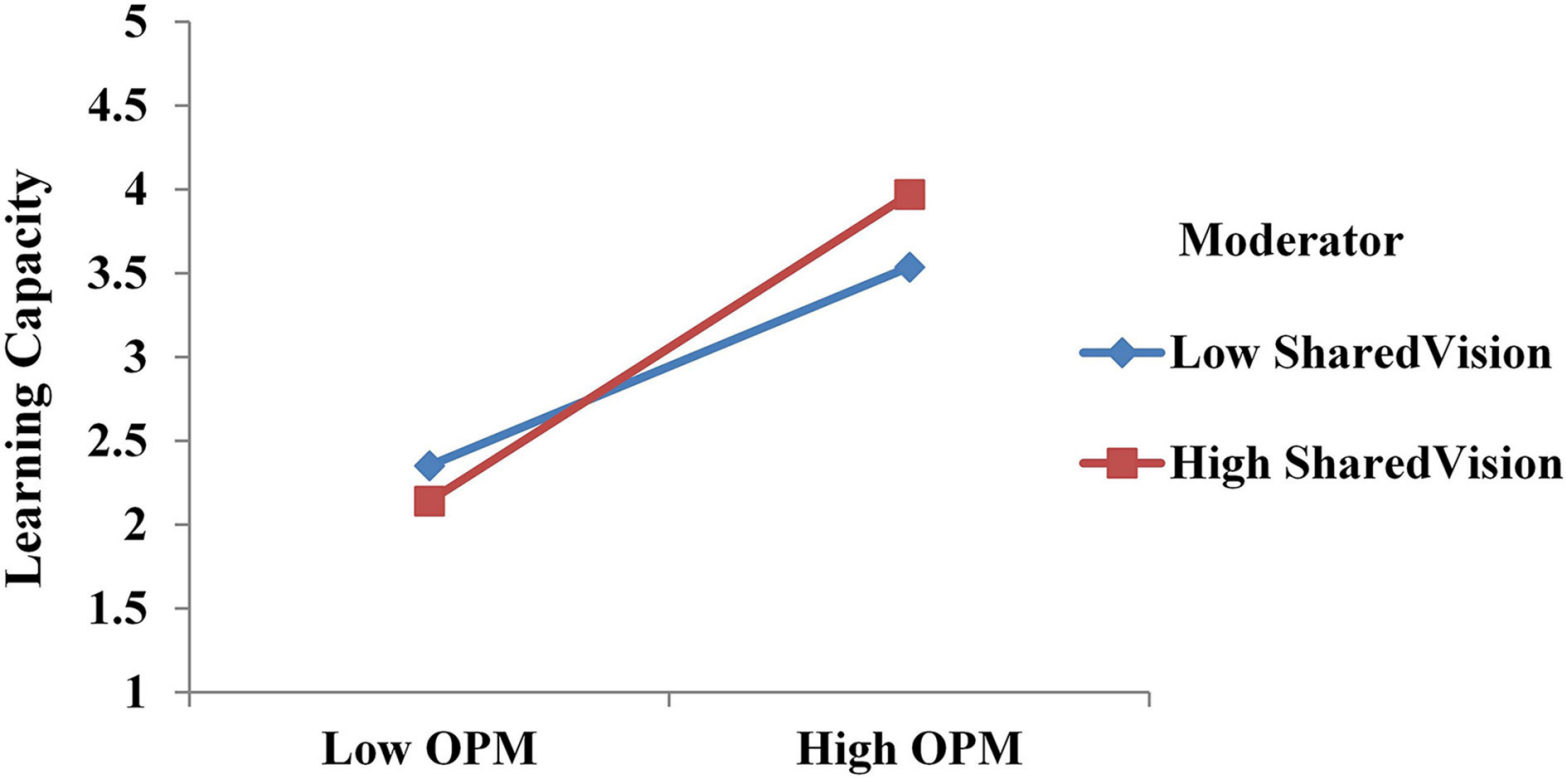
**Interaction effect**.

Hypothesis 2 is also supported in that SHV strengthens the positive effect of OPM on learning capacity. This can be shown in Figure 3. When SHV is high, the slope of the relationship between OPM and LCAP is steeper; and when SHV is low, the line is flatter. The standardized regression weight between the interaction variable (OPM X SHV) and the dependent variable (LCAP) is positive and significant at the 0.001 level. In sum, SHV moderates the effect of OPM on LCAP by strengthening the positive relationship.

## Model fit

Model fit is excellent as shown in Table 3 along with the “ideal thresholds” outlined by Hu and Bentler (1999). All thresholds are met. R-squared is also excellent at 73.4%; this reveals how much of the variance in the dependent variable is explained by the predictors.

**Table 3:** **Fit statistics for structural model**.

**Metric**	**Observed value**	**Ideal threshold**
CMIN/df	1.467	Between 1 and 3
CFI	0.993	>0.950
RMSEA	0.053	<0.060
PCLOSE	0.409	>0.050
SRMR	0.043	<0.080

## Other findings

Our survey also collected data on the degree of diverse investment expertise among committee members; this refers to the breath of investment expertise across various asset classes (such as domestic equities, international equities, fixed income, real estate, hedge funds and private equity). In the previous study (Lord, 2014b), diverse investment expertise was found to contribute both to knowledge acquisition and to knowledge implementation. This finding was in alignment with theory by Cohen and Levinthal (1989, 1990) that prior experience is a key determinant of absorptive (or learning) capacity. For this paper we employ one item representing diverse investment expertise and divide the respondents into two roughly equal groups. Group 1 consists of the 88 respondents who answered either “very strongly agree” or “strongly agree” to the following statement: “Our committee over the five-year period always included expertise across a broad variety of asset classes.” Group 2 consists of 80 respondents who answered either very strongly disagree, strongly disagree, somewhat disagree, neutral, or somewhat agree. In Table 4 we can see the differences in the mean scores between the two groups for shared vision, OPM and learning capacity.

**Table 4:** **Differences in groups based on levels of diverse investment expertise**.

	**DivExpGRPS**	***N***	**Mean**	**Std. deviation**	**Std. error mean**
SHV mean	1.00	88	6.4403	0.59692	0.06363
	2.00	80	5.8844	1.04786	0.11715
OPM mean	1.00	88	5.8665	0.76183	0.08121
	2.00	80	4.8656	1.16308	0.13004
LCap mean	1.00	88	5.7551	0.72600	0.07739
	2.00	80	4.7111	0.98236	0.10983

Using the independent samples *t*-test, there was a significant difference in the mean scores for the two groups across all three factors in the study; significance was at 0.001 for each factor and degrees of freedom were 166. The following *t* values were reported for each variable: shared vision, 4.273; OPM, 6.654; and learning capacity, 7.880. In sum, committees that had more diversified investment expertise across asset classes had higher levels of shared vision, OPM and LCAP than committees with less diversified expertise across asset classes. Therefore, committees wanting to increase their levels of shared vision, OPM and LCAP may want to consider diversifying the types of expertise on the committee. In the current research, the expertise that was examined all related to the broad realm of investments but it included variety of expertise within that realm.

In addition, our survey collected data on the degree of portfolio diversification across different asset classes, as discussed in a previous study (Lord, 2014b). By dividing our sample into two halves—one with the most diversified portfolios and the other with the least diversified portfolios—we found that the halves differ significantly with regards to the three variables in this study. For OPM and learning capacity, differences in the mean responses between more diversified portfolios and less diversified portfolios were significant at the 0.001 level. And for shared vision, the difference between more and less diversified portfolios was significant at the 0.01 level. In sum, more diversified investment portfolios occurred in committee environments of higher shared vision, OPM and learning capacity. In the previous paper (Lord, 2014b), portfolios with greater diversification among asset classes had higher risk-adjusted returns relative to their peers of similar size over a five-year period.

## Discussion

The findings in this study provide strong support to learning theorists' belief that OPM (also referred to as critical assessment, authentic inquiry or reflection) is a key determinant of learning capacity. In addition, the study is novel in finding that SHV has a positive and significant effect on the relationship between OPM and learning capacity. It is important to keep in mind that OPM in this study has a greater impact on LCAP than does shared vision. The co-existence of SHV and OPM in the model's configuration produces a greater effect on LCAP than OPM alone. In our view, that's because SHV not only provides direction and motivation for the group's efforts but also because its moderating effect is on an already-strong learning environment. If, as mentioned previously, closed-mindedness were hypothesized to reduce learning capacity, SHV could be expected to augment that effect. Therefore, consideration must be given to the context or environment in which SHV exists. In extreme situations, SHV could be used in studies with horrific results. Consider a model where “Hatred of Jews” contributed to “Deaths at Auschwitz.” It would seem logical to assume that “shared vision” among Hitler and his cronies would augment the relationship between “Hatred of Jews” and “Deaths at Auschwitz.” Happily, SHV in the current study co-exists with an independent and a dependent variable that are dramatically more positive.

Another important consideration is that SHV should not connote rigidity of the group's beliefs or goals. Especially in an environment with strong OPM and learning capacity, group members could be expected to re-examine their existing beliefs and goals, and to be willing to alter them based on greater understanding of the context in which they operate. OPM would essentially dictate an ongoing assessment of the group's purpose and goals to determine whether they are still justified.

This study also contributes to the literature on group conflict in that previous research focused on the benefits of task conflict to decision quality (Jehn, 1995; Amason, 1996) while this study links task conflict (as represented by OPM) to learning capacity. We believe it is quite likely that OPM (due to its similarities to task conflict) could also be found to have a positive effect on decision quality. One could easily argue that there is a strong correlation between those two outcomes. One might hypothesize, for example, that LCAP is an antecedent to decision quality. Our findings also support research positing that task conflict is very different from relationship (or affective) conflict in that the former is focused on the content of the task while the latter is focused on personal factors (Todorova et al., 2014; Weingart et al., 2014). Relationship or affective conflict can include interpersonal criticism, individual bragging, blaming, and defensiveness—all behaviors that can inhibit group learning; these types of behavior may occur in competitive environments where the “we” is superseded by the “me.” In contrast, cognitive or task conflict is oriented toward the substance of the work and helps to reveal additional insights and perspectives that contribute to group learning. In our view, group conflict that remains task oriented could be more accurately and positively framed as “productive disagreement” rather than “task conflict.”

In a recent addition to research on task conflict, Todorova et al. (2014) differentiate between *mild* and *intense* task conflict *expression*. Mild task conflict expression occurs when team members debate about differing ideas or opinions, and express different viewpoints about work issues. On the other hand, intense task conflict expression occurs when members criticize each other's viewpoints, clash about objectives/goals, and argue about desired output. While it is possible for both of these expressions of task conflict to remain focused on tasks, only *mild* task conflict expression had a significant, positive effect on information acquisition in their study. *Intense* task conflict expression, on the other hand, had a significant negative effect on information acquisition. The authors suggest that frequent, intense task conflict expressions can interfere with potential informational benefits since the intensity of arguments may limit information sharing and processing. We suggest that the OPM construct in our study is very similar to *mild* task conflict expression, and that it supports the findings of Todorova et al. (2014) that mild task conflict expression contributes significantly to learning. We concur that *intense* task conflict expression starts to resemble relationship conflict, which tends to detract from learning.

As for concerns about conformity, we contend that a group climate of OPM would be negatively related to compliance behaviors that have been associated with groupthink. In addition, SHV represents group members' *genuine* belief that they are working collaboratively toward a common purpose whereas conformity often represents situations where group members publicly “act” as though they are in agreement when, instead, they privately disagree. When beliefs are genuine they are internalized, whereas when expressions of belief are not genuine they may indicate compliance (McCauley, 1989).

While we are open to the view that SHV falls within the spectrum of cohesiveness, we would argue that the very strong influence of OPM in this study severely limits the possibility of the type of intense interpersonal cohesiveness that is associated with groupthink. In our view, groupthink is simply not compatible with either OPM or learning capacity. If group members are open-minded they are not consensus seeking for the sake of seeking consensus. In addition, if they are open-minded they want to seek new external information, to assimilate it and to apply it rather than conform to the stated group view without engaging in learning behaviors. There may be some degree of interpersonal cohesiveness built through the collective work of developing shared vision, and it could be argued that the cohesiveness around SHV may become so strong that it veers toward a group desire to be unanimous in thoughts and perspectives. In response, we offer a counterargument from this study's results that the concurrent presence of OPM–with its focus on critical assessment–will ward off that occurrence, just as we argue that SHV provides a curb on dialogs that may start out as open-minded but become so emotionally intense that they destroy the conditions and capacity for learning. In a sense, SHV and OPM may serve to regulate each other in healthy ways.

## Limitations and future directions

Our survey was conducted of “key informants” of college and university endowments, whereas multiple responses of members from each endowment committee likely would have been more representative. In addition, given that all respondents were associated with university endowments, the study may not be generalizable to other decision-making committees or boards.

While the methodology in this study employs a one-directional causal model, with OPM and the interaction variable (OPM combined with shared vision) leading to learning capacity, we believe it is more appropriate to consider the variables as reciprocal in that relationships can go in both directions. For example, it seems logical to believe that greater LCAP could lead to greater OPM, in that more implementations of learning would provide more instances for critical reflection. In addition, more OPM and the greater understanding associated with it could augment the group's SHV about its purpose and goals. And, as noted previously, we believe that OPM could help the group refine or even adopt a new SHV if it can no longer justify the old one. In short, the variables appear to be contemporaneously intertwined.

Another possible limitation is that we did not test or control for demographic factors such as ethnicity or gender; such inclusion could have enlightened our understanding of generalizability. Also, our construct for LCAP is unidimensional whereas a multi-dimensional construct could have provided more insights regarding how OPM and the interaction variable would influence each of the learning dimensions.

In addition, the study could have provided further insights if it had included a construct for interpersonal cohesiveness; this would have permitted us to contrast the influence of SHV vs. the influence of strong interpersonal cohesiveness on the relationship between OPM and learning capacity. The personality trait called “agreeableness” might be a starting place in considering a measure.

Future research could provide further insights into conditions for greater LCAP by addressing some of the limitations noted above as well as considering factors such as leadership styles and other facets of a learning environment.

## Conclusion

We believe this study provides new insights about group dynamics that affect collective learning. By employing SHV as a moderator of the effect of OPM on group learning capacity, the study makes an innovative contribution to other research that encompasses both SHV and OPM. Authors Amason and Sapienza (1997) discuss the need for both openness and mutuality in effective team decision-making. They define mutuality as the degree to which team members share goals and responsibilities, and openness as the team's “propensity to tolerate, encourage, and engage in open, frank expression of views.” Thus, “mutuality” is related to “shared vision,” and “openness” is related to “OPM.” Researchers stress the importance of getting the balance right (Jehn, 1995; Amason and Sapienza, 1997). If there is too much mutuality and not enough cognitive conflict (or OPM), group members may become complacent or agree too readily such that LCAP and decision quality suffer. However, if the openness becomes so heated that it resembles *intense* task conflict expression, the effects can include confusion, personal conflict and even closed-mindedness, all of which would detract from learning.

In conclusion, we would argue that there's some truth to Oscar Wilde's quote: “Everything in moderation, including moderation.” A proper balance between OPM and SHV appears to offer true benefits such as greater learning capacity. On the other hand, there also may be truth to another quote by Wilde: “Moderation is a fatal thing. Nothing succeeds like excess.” With regards to the latter, excess LCAP may contribute to success. No doubt, one must choose his/her excesses carefully.

### Conflict of interest statement

The author declares that the research was conducted in the absence of any commercial or financial relationships that could be construed as a potential conflict of interest.

## Appendix: constructs, definitions and items

### Open-mindedness (OPM)

The committee's critical assessment of its assumptions, beliefs and prior actions, as well as its openness to new ideas.

1. Committee members routinely judged the quality of the decisions they made.
2. The committee was not afraid to reflect critically on investment-related assumptions it made.
3. Committee members realized that the way we perceive the markets must be continually questioned.
4. Committee members routinely made critical assessments of the investment approach.

### Shared vision (SHV)

A common mental model for the direction of the organization.

1. Our committee was in agreement about the endowment's purpose.
2. Committee members were committed to the goals for the endowment.
3. There was agreement among committee members about the vision for the endowment.
4. Committee members viewed themselves as partners in our efforts for the endowment.

### Learning capacity (LCAP)

The committee's ability to acquire, assimilate and implement knowledge successfully.

1. The committee collected in-depth information that was relevant to our investment decisions.
2. The committee quickly recognized shifts in the financial markets.
3. The committee quickly analyzed and interpreted changing market conditions.
4. The committee quickly determined the usefulness of new investment-related knowledge to existing knowledge.
5. The committee was capable of assessing potential investment opportunities based on its existing knowledge.
6. The committee knew how to implement new investment knowledge.
7. The committee had routines in place that it believed are essential for superior long-term performance.
8. The committee had policies in place that it believed are essential for superior long-term performance.
9. The committee knew how to capitalize on its investment knowledge.
